# Knowledge and Attitude of Dentists Toward Geriatric Patients: A Systematic Review and Meta-Analysis

**DOI:** 10.7759/cureus.48339

**Published:** 2023-11-06

**Authors:** Rawa Abdelrahim, Sarah Salah Gaafar, Khuthija Khanam, Malak Albalawi

**Affiliations:** 1 Department of Preventive Dental Sciences, Dar Al Uloom University, Riyadh, SAU; 2 Department of Restorative and Prosthetic Dental Sciences, College of Dentistry, Dar Al Uloom University, Riyadh, SAU; 3 Department of Restorative and Prosthetic Dental Sciences, Dar Al Uloom University, Riyadh, SAU; 4 Department of Preventive Dental Sciences, College of Dentistry, Dar Al Uloom University, Riyadh, SAU

**Keywords:** meta-analysis, systematic review, geriatric patients, dentists, attitude, knowledge

## Abstract

The knowledge and attitude of dentists toward geriatric patients are crucial for providing optimal oral healthcare to this growing population. This systematic review and meta-analysis aimed to examine the association between dental professionals’ experience and their knowledge and attitude scores toward geriatric patients. A comprehensive search was conducted across multiple databases to identify relevant epidemiological and cross-sectional studies. The selected studies underwent a rigorous screening process, and data extraction was performed systematically. The Assessment of the Methodological Quality of Systematic Reviews tool was used to assess the risk of bias in the included studies. Three studies were ultimately selected for further analysis. For dental professionals with less than five years of experience, no significant association was found between knowledge scores and experience (odds ratio (OR) = 1.00, 95% confidence interval (CI) = 0.71 to 1.41). However, dental professionals with 5 to 10 years, greater than 10 years, and greater than 15 years of experience showed a statistically significant association between knowledge scores and experience (OR = 0.61, 95% CI = 0.48 to 0.77; OR = 0.69, 95% CI = 0.54 to 0.89; OR = 0.59, 95% CI = 0.44 to 0.79, respectively). The overall analysis indicated a significantly positive association between knowledge scores and experience among dental professionals (OR = 0.68, 95% CI = 0.59 to 0.78). The findings provide evidence of a positive association between dental professionals’ experience and their knowledge and attitude scores toward geriatric patients. The findings highlight the importance of experience in enhancing dental professionals’ understanding and approach to geriatric dental care. These results have implications for improving dental education, training, and policy development to better address the oral health needs of older adults.

## Introduction and background

The geriatric population presents distinct oral healthcare needs that require special attention from dental professionals [1]. As people age, they are more susceptible to a range of oral health conditions and challenges that can significantly impact their overall well-being and quality of life [2]. Understanding these unique needs is crucial for providing effective oral healthcare and maintaining the oral health of older adults [3].

One of the key oral health concerns among geriatric individuals is the increased prevalence of chronic conditions such as periodontal disease, tooth loss, dry mouth (xerostomia), and oral cancer [4]. These conditions can be influenced by a combination of age-related factors, systemic health conditions, medications, and lifestyle habits [5]. As a result, older adults may experience a higher risk of oral infections, difficulty in chewing and swallowing, impaired speech, and compromised nutrition [6].

Geriatric dentistry encompasses various aspects, including age-related changes in oral health, the impact of systemic diseases on oral health, and the management of oral conditions in older adults [6]. Dentists need to be equipped with up-to-date knowledge about these factors to provide appropriate preventive, diagnostic, and therapeutic interventions [7]. This includes awareness of common oral health conditions prevalent in geriatric patients, such as periodontal diseases, tooth loss, dry mouth, and oral cancer [8]. Additionally, dentists should have a comprehensive understanding of the physiological, psychological, and social aspects that influence oral health in older adults [8].

Moreover, the attitude of dentists toward geriatric patients significantly affects the delivery of care. A positive and empathetic attitude promotes effective communication, instills confidence, and enhances patient satisfaction [9]. Geriatric patients often face physical and cognitive limitations that may hinder their ability to receive dental treatment comfortably. Dental professionals with a positive attitude are more likely to accommodate these challenges, adapt their techniques, and provide individualized care tailored to the unique needs and preferences of older adults [10].

Moreover, addressing the psychological and social aspects of oral healthcare is crucial for geriatric patients [11]. Dentists and dental healthcare providers should foster a compassionate and patient-centered approach, ensuring effective communication, understanding, and respect for the preferences and concerns of older adults [11]. This includes creating a comfortable and accessible dental environment, providing clear explanations of treatment options, and involving patients in decision-making processes [12].

However, assessing the knowledge and attitude of dentists toward geriatric patients is a complex endeavor. Factors such as dental education, continuing professional development, exposure to geriatric patients during training, and personal experiences can influence dental professionals’ perspectives. Therefore, this systematic review and meta-analysis aimed to synthesize the available evidence on the knowledge and attitude of dentists toward geriatric patients. By examining multiple studies in a systematic and comprehensive manner, this study aimed to provide a comprehensive overview of the current understanding in this area and identify potential gaps in knowledge by employing a statistical approach using a meta-analysis.

## Review

Review design and protocol

**Figure 1:**
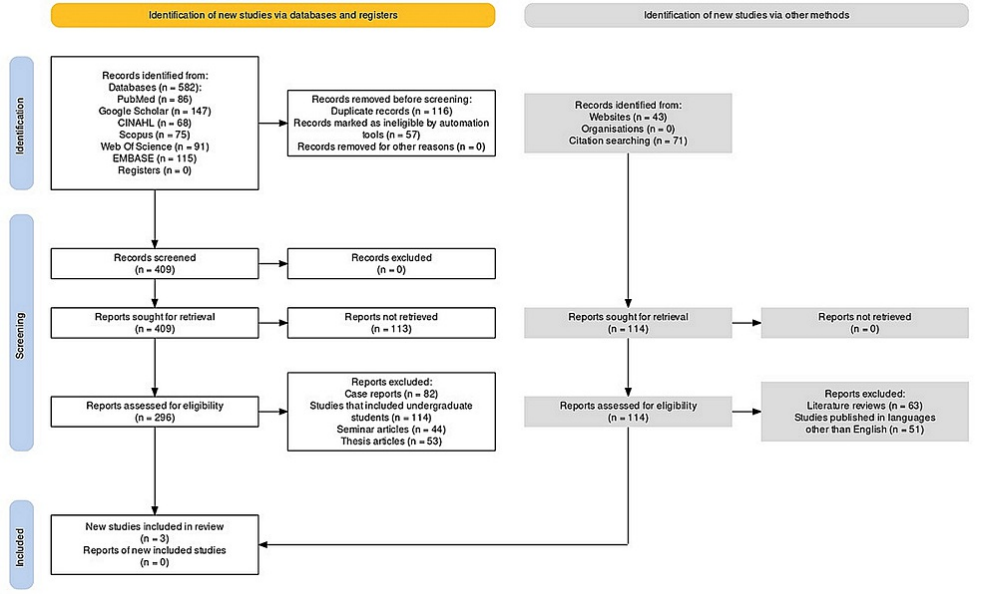
PRISMA protocol employed in the study. CINAHL: Cumulative Index of Nursing and Allied Health Literature; EMBASE: Excerpta Medica Database; PRISMA: Preferred Reporting Items for Systematic Reviews and Meta-Analyses

The Preferred Reporting Items for Systematic Reviews and Meta-Analyses (PRISMA) protocol [13,14] was employed to guide this investigation. The first step in utilizing this protocol involved clearly defining the research question and establishing inclusion and exclusion criteria for the selection of relevant studies (Figure 1). The utilization of this protocol further ensured a systematic and transparent approach to this systematic review and meta-analysis. Adhering to the PRISMA guidelines facilitated the rigorous selection and evaluation of studies, standardized data extraction, and transparent reporting of the findings. By following the PRISMA protocol, this study enhanced the reliability, credibility, and reproducibility of the statistical findings obtained through this review (Figure 1).

The PICOS strategy ensured that the review addressed the specific aspects of interest related to the knowledge and attitude of dentists toward geriatric patients.

The “Population” component focused on identifying the target population of interest, which in this case was dentists. Specifically, the review aimed to include studies that investigated the knowledge and attitude of dental professionals toward geriatric patients. This population encompassed both general dentists and specialized dental practitioners.

The “Intervention” component referred to the key factor or exposure being assessed in the included studies. In this review, the primary intervention of interest was the knowledge and attitude of dentists toward geriatric patients. The review aimed to explore the level of knowledge and the attitude exhibited by dental professionals regarding geriatric dental care.

The “Comparison” component considered any relevant comparator groups or alternative interventions. In this review, the focus was on comparing the knowledge and attitude of dentists toward geriatric patients across different demographic factors, such as age, gender, professional experience, and geographic region. The aim was to identify any variations or disparities in knowledge and attitude based on these factors.

The “Outcome” component specified the desired outcomes or endpoints that were assessed in the included studies. In this review, the primary outcomes of interest were the knowledge level and attitude of dentists toward geriatric patients. This encompassed various aspects, including knowledge about oral health issues in the elderly, familiarity with geriatric-specific dental procedures, attitudes toward treating geriatric patients, and perceptions of barriers or challenges in providing dental care to older individuals.

Finally, the “Study Design” component indicated the types of study designs eligible for inclusion in the review. The review included both observational and cross-sectional studies that examined the knowledge and attitude of dentists toward geriatric patients. This design choice allowed for a comprehensive evaluation of the current evidence available on the topic.

Search protocol

The database search strategy employed in this investigation aimed to identify relevant epidemiological and cross-sectional studies. The strategy involved systematic and comprehensive searching of multiple electronic databases to ensure a thorough retrieval of eligible studies. Six different online databases were consulted for selecting the relevant articles. The search terms used were carefully selected to capture relevant studies related to the knowledge and attitude of dentists toward geriatric patients. The keywords and Medical Subject Headings (MeSH) terms included variations of “dentists,” “geriatric patients,” “knowledge,” “attitude,” and “cross-sectional studies.” The search strategy was adapted and tailored to each specific database using appropriate syntax and operators, such as Boolean operators (AND, OR), wildcards, and truncation, as shown in Table 1.

**Table 1:** Search strings utilized across the assessed databases.

Database	Search string
PubMed	(“Dental Professionals”[Title/Abstract] OR “Dentists”[Title/Abstract]) AND (“Geriatric Patients”[Title/Abstract] OR “Older Adults”[Title/Abstract]) AND (“Knowledge”[Title/Abstract] OR “Attitude”[Title/Abstract] OR “Experience”[Title/Abstract])
Embase	(‘Dentists’:ab,ti OR ‘Dental professionals’:ab,ti) AND (‘Elderly’:ab,ti OR ‘Aged’:ab,ti) AND (‘Knowledge’:ab,ti OR ‘Attitude’:ab,ti OR ‘Experience’:ab,ti)
Scopus	(TITLE-ABS-KEY (dentists) OR TITLE-ABS-KEY (dental health professionals)) AND (TITLE-ABS-KEY (older adults) OR TITLE-ABS-KEY (elderly)) AND (TITLE-ABS-KEY (knowledge) OR TITLE-ABS-KEY (attitude) OR TITLE-ABS-KEY (experience))
Web of Science	(TI=(Dentists) OR TI=(Dental Health Professionals)) AND (TI=(Elderly Patients) OR TI=(Aged)) AND (TI=(Knowledge) OR TI=(Attitude) OR TI=(Experience))
PsycINFO	(AB (Dental Health Professionals) OR AB (Dentists)) AND (AB (Geriatric Population) OR AB (Aged)) AND (AB (Knowledge) OR AB (Attitude) OR AB (Experience))
Cochrane Library	(Title, Abstract, Keywords: Dentists OR Dental Health Professionals) AND (Title, Abstract, Keywords: Elderly OR Senior citizens) AND (Title, Abstract, Keywords: Knowledge OR Attitude OR Experience)

Selection criterion

The inclusion and exclusion criteria were designed to ensure the selection of relevant studies while maintaining a focus on epidemiological and cross-sectional designs.

Inclusion Criteria

Study Design: Only epidemiological and cross-sectional studies were included in this review, as they provide valuable insights into the knowledge and attitude of dentists toward geriatric patients.

Participants: The study participants were limited to practicing dentists, including clinicians and academicians.

Outcome of interest: The primary outcome of interest was the knowledge and attitude of dentists toward geriatric patients.

Assessment technique: Studies that utilized validated questionnaires or assessment tools to measure knowledge and attitude were included.

Language: Studies published in English were considered for inclusion.

Exclusion Criteria

Study design: Any study design other than epidemiological or cross-sectional was excluded from this review.

Participants: Studies involving undergraduate dental students were excluded from the analysis. This decision was justified by the focus on assessing the knowledge and attitude of practicing dentists, as their experience and exposure to geriatric patients may differ significantly from that of undergraduate students.

Non-English studies: Studies published in languages other than English were excluded due to potential limitations in language translation and interpretation.

The exclusion of studies involving undergraduate dental students enhances the credibility of this systematic review and meta-analysis by narrowing the focus to practicing dentists who have clinical experience and professional exposure to geriatric patients. Undergraduate students are at an early stage of their dental education and may not have substantial practical experience in dealing with elderly patients. By excluding studies involving undergraduate dental students, the review ensures a more accurate representation of the knowledge and attitude of experienced dentists, which is crucial for providing reliable and applicable findings in the context of geriatric dental care.

Data extraction and reviewer protocol

A meticulous and standardized data extraction strategy was implemented for this review. The process involved several key steps to ensure the collection of relevant and reliable information from the selected studies. First, a data extraction form was developed, encompassing predetermined variables and pertinent details to be extracted from each study. This form served as a structured template to gather essential study characteristics and outcomes related to the knowledge and attitude of dentists toward geriatric patients. Following the form’s development, a pilot testing phase was conducted on a subset of included studies. This step allowed for the refinement and enhancement of the data extraction form, ensuring its clarity, comprehensiveness, and ability to capture the required information accurately. To maintain objectivity and minimize potential biases, two independent reviewers performed the data extraction process. Each reviewer independently extracted the data from the selected studies using the standardized data extraction form. Any disagreements or discrepancies between the reviewers were resolved through discussion and consensus. The data extraction form included variables such as study characteristics (e.g., author, year, location), participant demographics (e.g., sample size, age, gender), study design, assessment techniques used to measure knowledge and attitude, and relevant outcome measures. Additionally, a predetermined quality assessment tool was applied during the extraction process to evaluate the methodological quality and risk of bias in each included study. Once the data extraction was completed, the extracted data were synthesized and analyzed. This involved summarizing the findings across studies, identifying common themes or patterns, and exploring any variations or inconsistencies among the included studies. The synthesis of data allowed for a comprehensive overview of the knowledge and attitude of dentists toward geriatric patients, contributing valuable insights to inform evidence-based decision-making and guide future research in this area. By employing this systematic and rigorous data extraction strategy, this review ensured the accurate and reliable collection of information from the included studies. The independent extraction by two reviewers and the incorporation of a quality assessment step enhanced the credibility and robustness of the data extraction process. Ultimately, the comprehensive analysis of the extracted data provided a comprehensive understanding of the knowledge and attitude of dentists toward geriatric patients, offering valuable implications for clinical practice, education, and further research in this field.

Assessment of bias

In this review, the Assessment of the Methodological Quality of Systematic Reviews (AXIS) tool [15,16] was employed to assess the risk of bias in the included studies. The AXIS tool is a comprehensive and structured protocol specifically designed for systematic reviews and meta-analyses to evaluate the methodological quality and potential biases in the selected studies. Using the AXIS tool, each study included in the review underwent a rigorous bias assessment process. The protocol involved assessing multiple domains that could influence the validity and reliability of the study findings. By employing the AXIS tool, the bias assessment process in this systematic review and meta-analysis ensured a comprehensive evaluation of the methodological quality and potential biases across multiple domains in the included studies. This systematic approach provided a robust framework for assessing the risk of bias and strengthening the validity and reliability of the review findings. Ultimately, the bias assessment using the AXIS tool enhanced the overall quality and credibility of the systematic review, contributing to evidence-based decision-making and providing valuable insights into the knowledge and attitude of dentists toward geriatric patients.

Statistical protocol

The meta-analysis was conducted using RevMan 5 (v5.4.1), which is a widely used software for performing statistical analyses in systematic reviews. The primary objective of the meta-analysis was to assess the significance of knowledge and attitude scores across different categories pertaining to the experience of dental professionals. To analyze the knowledge scores, the odds ratio (OR) and risk ratio (RR) were used as effect measures, representing the association between the experience categories and knowledge scores. A finite element (FE) model was employed for the meta-analysis, assuming that the true effect size was the same across all studies included in the analysis. By using RevMan 5, the data from the selected studies were extracted and combined to calculate the pooled OR and RR estimates, along with their corresponding confidence intervals (CIs). The FE model incorporated the individual effect estimates from each study and generated an overall estimate of the effect size, providing a summary measure of the association between the experience categories and knowledge scores. This meta-analysis protocol allowed for a comprehensive and quantitative synthesis of the findings across the included studies, enabling a better understanding of the relationship between the experience of dental professionals and their knowledge and attitude scores regarding geriatric patients. The utilization of RevMan 5 and the FE model ensured a standardized and rigorous approach to the meta-analysis, enhancing the reliability and robustness of the results.

**Figure 2:**
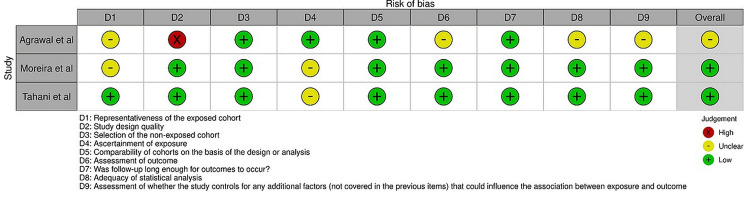
Bias assessment of the studies selected for this review. Agrawal et al. [17]; Moreira et al. [18]; Tahani et al. [19].

Figure 2 presents the results of bias assessment across the three selected studies [17-19] using the AXIS tool.

The AXIS tool evaluates multiple domains related to bias in each study. Regarding study design quality, one study was categorized as a “high” risk of bias, suggesting a higher likelihood of bias due to potential limitations in the study design. The other two studies were classified as a “low” risk of bias, indicating a lower likelihood of bias based on the study design. The ascertainment of exposure was evaluated as a “low” risk of bias in most cases, indicating robust methods for determining exposure status. However, one study received an assessment of “unclear,” suggesting a lack of clarity or potential limitations in assessing exposure. The assessment of outcome was generally categorized as a “low” risk of bias, indicating reliable methods for evaluating the outcomes of interest in the studies. The adequacy of statistical analysis was generally categorized as “low” across all studies. Lastly, the assessment of whether the studies controlled for additional factors that could influence the association between exposure and outcome was generally classified as “unclear” or “low” risk of bias, indicating a lack of clarity or potential limitations in controlling for these factors. Overall, the selected studies demonstrated a low level of risk of bias across the assessed domains, highlighting the importance of considering potential limitations and sources of bias when interpreting their findings.

Table 2 provides information on the selected studies [17-19], including the study ID, year of publication, region of study, sample size (n), mean age in years, and gender ratio.

**Table 2:** Assessment of the demographic variables of the selected studies. Study ID: study identification

Study ID	Year	Region	Sample size (n)	Mean age (in years)	Gender ratio
Agrawal et al. [17]	2020	India	294	Unspecified	146 males
Moreira et al. [18]	2011	Brazil	276	Unspecified	131 males
Tahani et al. [19]	2021	Iran	231	34.4 ± 8.1	86 males

The studies were conducted in India, Brazil, and Iran, and each study focused on a different population of interest. In terms of sample size, the studies included 294, 276, and 231 participants, respectively. However, the mean age of the participants varied across the studies. While the mean age was unspecified in two studies, the third study reported a mean age of 34.4 years with a standard deviation of 8.1 years. This suggests that the populations under investigation represented a range of age groups, albeit with different degrees of precision in the reported age information. Regarding the gender distribution, the studies provide insights into the male-to-female ratio within each sample. The first study reported 146 males without specifying the number of females, while the second study indicated 131 males. In the third study, 86 out of 231 participants were males. These gender ratios highlight the presence of gender diversity within the study populations but may also indicate potential gender imbalances in certain studies.

Table 3 provides insights into the selected studies [17-19], presenting information on the study ID; protocol; primary study objective; groups assessed; knowledge, attitudes, and practice (KAP) assessment technique; assessment of knowledge scores among professionals; and the observed inferences.

**Table 3:** Assessment of geriatric variables and their associated inferences in the selected studies. KAP: knowledge, attitudes, and practices; SDA: secondary data analysis; Study ID: study identification

Study ID	Protocol	Primary study objective	Groups assessed	KAP assessment technique	Assessment of knowledge scores amongst professionals	Inference observed
Agrawal et al. [17]	Cross-sectional	SDA in elderly	Clinicians, academicians, and both	Questionnaire	Only 22% of the assessed sample size had positive KAP toward SDA	Despite having a favorable attitude toward the SDA concept and just a small number of dentists using it, the majority of the dentists were unaware of it
Moreira et al. [18]	Cross-sectional	General dental treatment in the elderly	Clinicians, academicians, and both	Questionnaire (Likert scale-based)	56 and 151 dentists showed high and moderate level of knowledge regarding elderly dental care, respectively	Women showed higher attitude scores toward the elderly, and graduates with experience in the public sector showed more expertise in handling elderly patients
Tahani et al. [19]	Cross-sectional	General dental treatment in the elderly	Clinicians, academicians, and both	Questionnaire (Likert scale-based)	In terms of knowledge concerning dental care for the elderly, almost 87% of participants exhibited moderate knowledge, while nearly 3% showed good knowledge	Almost 30% of the dentists acknowledged feeling inadequately prepared to treat older patients, and 40% of the dentists believed that the current dental education provided in dental schools did not offer sufficient training in this regard

The studies were all cross-sectional in nature and aimed to investigate aspects related to general dental treatment in the elderly population. Various groups, including clinicians, academicians, or both, were assessed in each study. In terms of the KAP assessment technique, a questionnaire was utilized in all three studies. The questionnaires employed different formats, such as Likert scale-based assessments, to evaluate the KAP of the dental professionals under investigation.

**Figure 3:**
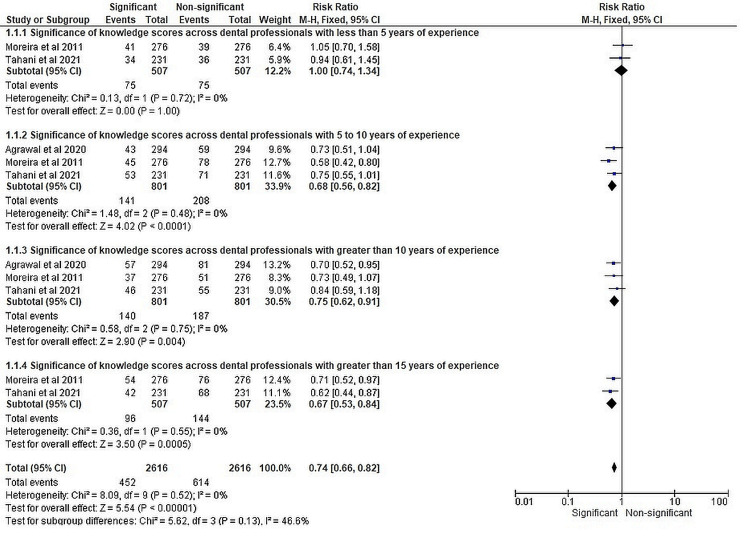
Significance of knowledge and attitude scores across different categories of experience of dental professionals represented in terms of RR. Agrawal et al. [17]; Moreira et al. [18]; Tahani et al. [19]. M.H: Mantel-Haenszel; CI: confidence interval; chi2: chi-squared; RR: risk ratio

The forest plot presented in Figure 3 displays the significance of knowledge and attitude scores across different categories of dental professionals based on their years of experience. For dental professionals with less than five years of experience, the pooled OR was 1.00, with a 95% CI of 0.71 to 1.41. The test for overall effect was not statistically significant (Z = 0.00, p = 1.00), indicating no significant association between knowledge scores and experience in this group. For those with 5 to 10 years of experience, the pooled OR was 0.61, with a 95% CI of 0.48 to 0.77. The test for overall effect was statistically significant (Z = 4.05, p < 0.0001), indicating a significant association between knowledge scores and experience. In the group with greater than 10 years of experience, the pooled OR was 0.69, with a 95% CI of 0.54 to 0.89. The test for overall effect was significant (Z = 2.91, p = 0.004), indicating a significant association between knowledge scores and experience. For dental professionals with greater than 15 years of experience, the pooled OR was 0.59, with a 95% CI of 0.44 to 0.79. The test for overall effect was statistically significant (Z = 3.53, p = 0.0004), indicating a significant association between knowledge scores and experience. Considering all the categories together, the overall pooled OR was 0.68, with a 95% CI of 0.59 to 0.78. The test for overall effect was highly significant (Z = 5.57, p < 0.00001), indicating a significant association between knowledge scores and experience across all categories. Heterogeneity was observed within each category, with I^2^ values ranging from 0% to 1%. The test for subgroup differences indicated no significant differences between the categories (Chi-square = 6.55, df = 3, p = 0.09), although there was moderate heterogeneity (I^2^ = 54.2%).

The forest plot in Figure 3 presents the analysis of the significance of knowledge and attitude scores across different categories of experience among dental professionals using the measure of RR.

The forest plot is divided into four experience categories, namely, less than five years, 5 to 10 years, greater than 10 years, and greater than 15 years. For dental professionals with less than five years of experience, the pooled RR was 1.00, with a 95% CI of 0.74 to 1.34. The test for overall effect was not statistically significant (Z = 0.00, p = 1.00), indicating no significant association between knowledge and attitude scores and experience in this group. For those with 5 to 10 years of experience, the pooled RR was 0.68, with a 95% CI of 0.56 to 0.82. The test for overall effect was highly significant (Z = 4.02, p < 0.0001), indicating a significant association between knowledge scores and experience. In the group with greater than 10 years of experience, the pooled RR was 0.75, with a 95% CI of 0.62 to 0.91. The test for overall effect was significant (Z = 2.90, p = 0.004), indicating a significant association between knowledge scores and experience. For dental professionals with greater than 15 years of experience, the pooled RR was 0.67, with a 95% CI of 0.53 to 0.84. The test for overall effect was highly significant (Z = 3.50, p = 0.0005), indicating a significant association between knowledge scores and experience. Across all categories, the overall pooled RR was 0.74, with a 95% CI of 0.66 to 0.82. The test for overall effect was highly significant (Z = 5.54, p < 0.00001), indicating a significant association between knowledge scores and experience across all categories. There was low heterogeneity within each category, with I^2^ values of 0%, indicating minimal variation between the studies. The test for subgroup differences indicated no significant differences between the categories (Chi-square = 8.09, df = 9, p = 0.52), and there was no substantial heterogeneity observed (I^2^ = 0%).

Discussion

The significance of the studies included in this analysis lies in their contribution to understanding the KAP of dental professionals regarding general dental treatment in the elderly population. By assessing these factors, the studies shed light on the current state of awareness and preparedness among clinicians and academicians in providing dental care to older patients. The findings reveal important insights and highlight areas that require attention and improvement. The results indicating a lack of awareness and knowledge among dental professionals about specific concepts or practices demonstrate the need for targeted educational interventions and training programs. Enhancing knowledge and promoting positive attitudes toward elderly dental care can help improve the quality of oral health services provided to this vulnerable population. Strategies such as incorporating geriatric dentistry modules into dental education curricula or offering continuing education programs focused on elderly dental care could be considered to bridge the identified knowledge gaps. Moreover, the studies’ identification of factors associated with higher levels of knowledge, such as gender and experience in the public sector, provides valuable information for designing interventions tailored to specific professional groups. Understanding these factors can aid in the development of targeted strategies to enhance knowledge and skills among dental professionals, ultimately leading to improved oral health outcomes for elderly patients. Regarding the non-inclusion of studies with undergraduate students, this exclusion enhances the credibility of the review by focusing specifically on dental professionals who have completed their training and are actively engaged in providing dental care. By excluding undergraduate students, who may not have the same level of expertise and experience as practicing professionals, the review maintains a higher level of relevance and applicability to the target population. This approach strengthens the reliability and validity of the findings, as the experiences and perspectives of professionals in active practice are likely to be more representative of the challenges and realities faced in providing dental care to the elderly. By limiting the analysis to studies involving dental professionals, the review can provide more specific and targeted recommendations for improving knowledge and practices in this professional group, thus maximizing the potential impact of interventions and initiatives aimed at enhancing elderly dental care.

Findings from the first study [17] revealed that only 22% of the assessed sample size had a positive KAP toward a specific concept, namely secondary data analysis. Despite a favorable attitude toward the concept and a small number of dentists utilizing it, the majority of dentists remained unaware of it. The second study [18] demonstrated that 56 dentists exhibited a high level of knowledge, while 151 dentists showed a moderate level of knowledge regarding elderly dental care. The study also observed that women had higher attitude scores toward the elderly, and graduates with experience in the public sector displayed more expertise in handling elderly patients. In the third study [19], the assessment of knowledge concerning dental care for the elderly revealed that almost 87% of the participants displayed moderate knowledge, while nearly 3% showed good knowledge. Additionally, the study found that around 30% of the dentists acknowledged feeling inadequately prepared to treat older patients, and 40% of the dentists believed that the current dental education provided in dental schools lacked sufficient training in this specific area. The information provided in Table 3 offers a glimpse into the findings of the selected studies, focusing on knowledge scores and related inferences. However, it is important to consider that these summaries provide only a fraction of the complete study results. For a comprehensive understanding, further analysis of the complete findings, methodology, and additional variables investigated in each study is necessary.

A previous study [20] reported that a significant proportion of dentists lacked awareness of geriatric dentistry, with almost 90% demonstrating poor knowledge and nearly 12% having moderate knowledge. However, in our study, the majority of participants displayed moderate knowledge, while a small percentage reported poor knowledge regarding geriatric dentistry. This disparity may be partially attributed to differences in the types and number of questions posed. Another study [21] investigating dental students’ knowledge and attitude toward geriatric dentistry found that the majority had low-to-moderate levels of knowledge.

A study conducted in Europe [22] revealed that one-fourth and one-third of dentists in the Netherlands and Belgium, respectively, reported having patient populations comprising individuals aged 65 and above. These discrepancies could be explained, in part, by variations in the extent of geriatric education provided by dental schools in Iran and Europe. It is well-known that dental schools providing ample clinical experience can instill greater confidence and comfort among dentists in treating older patients [23].

An evaluation of geriatric dentistry education in European dental schools [24] demonstrated that a majority of universities offered dedicated modules and specialized courses for older individuals. In contrast, our study revealed a high percentage of dentists who found the current training received during dental school inadequate. Another study focused on dental schools in Iran [25] supported these findings, indicating insufficient courses and educational time allocated to geriatric education. Among the surveyed schools, nearly 73% had incorporated an elderly dental education module, but only one-eighth of those had conducted group seminars or occasional lectures. Furthermore, half of the schools provided clinical education in geriatric dentistry, while none had a separate department or specialized clinic for geriatric dentistry or a referral system for older patients from hospitals to dental faculties.

The relationship between knowledge acquisition and attitude shift among dental students is intricate. While dental students can acquire knowledge of aging through academic courses, the direct correlation between knowledge gain and attitude change is not straightforward [26]. In our study, a weak but significant correlation between knowledge and attitude was observed. Various discipline-specific interventions, such as aging awareness training, multi-modal interventions, clinical geriatric rotations, and senior mentoring programs, have been employed to positively shape and change attitudes [27]. Factors such as personal experience with older individuals and societal influence have been identified as key predictors of attitudes toward this age group [28-30]. Professional socialization, including exposure to faculty members with positive attitudes toward older adults, can also play a role in fostering positive attitudes among dental students [31]. However, a survey conducted at a university [32] revealed that significant changes occurred in dental students’ feelings toward treatment and willingness to treat underserved populations, including the elderly, five years after graduation. Initially, they held more negative attitudes toward treating low-income patients and the frail elderly. This suggests that variables beyond exposure to patients may influence students’ willingness to treat underserved populations.

Regarding barriers to access to dental care, a study [33] categorized cost, fear, availability, accessibility, and characteristics of the dentist as the main active barriers for older individuals. In another recent investigation [34] conducted during the COVID-19 pandemic, it was discovered that even after adhering to all of the COVID-19 practice standards, 70% of physicians still found it challenging to provide dental treatment to elderly patients. Dental professionals should take extra care when treating older patients who have COVID-19-related issues, according to about one-third of dentists. Therefore, more research on post-COVID-19 sequelae is necessary, as are guidelines for caring for the elderly throughout the epidemic.

As the geriatric population continues to grow globally, it is imperative to prioritize geriatric oral healthcare in dental education, professional training, and healthcare policies [35]. Dental professionals should receive comprehensive training in geriatric dentistry, including knowledge of age-related oral health changes, specific treatment considerations, and strategies for managing the unique needs of older adults [36]. Interdisciplinary collaboration with other healthcare providers, such as geriatricians and primary care physicians, is also vital for holistic care [37]. Promoting preventive oral healthcare is paramount in managing the oral health of the geriatric population [38]. Regular dental check-ups, oral hygiene instruction, and preventive interventions such as fluoride treatments and dental sealants can help mitigate the risk of oral diseases and maintain oral health. Early detection and treatment of oral conditions are vital to prevent their progression and minimize potential complications.

Several limitations need to be acknowledged regarding this study. First, the analysis relied on a limited number of selected studies, which may restrict the generalizability of the findings to a broader population of dental professionals. The inclusion of more diverse studies from various regions and settings would have provided a more comprehensive understanding of the KAP regarding elderly dental care. Another limitation pertains to the cross-sectional nature of the included studies. Cross-sectional designs provide a snapshot of information at a specific time point but do not establish causality or allow for the assessment of temporal relationships. Therefore, the findings regarding KAP should be interpreted cautiously, as they represent associations rather than causal effects. Furthermore, the studies relied primarily on self-report measures, such as questionnaires, which are subject to response biases and social desirability effects. The reliance on self-assessment may introduce inaccuracies or overestimations of knowledge and practices, leading to potential measurement bias in the findings. Additionally, the lack of detailed demographic information in some studies, such as socioeconomic status or years of professional experience, limits the ability to explore potential confounding factors or assess their influence on the outcomes. This omission hinders a comprehensive understanding of the factors that may contribute to the observed KAP among dental professionals.

## Conclusions

This investigation has revealed a significant and positive relationship between the experience of dental professionals and their knowledge and attitude scores toward geriatric patients. The findings underscore the crucial role that experience plays in improving dental professionals’ understanding and approach to geriatric dental care. These results have important implications for dental education, training, and policy development, aiming to enhance the provision of oral healthcare for older adults. By enhancing dental education, training, and policy initiatives, it is possible to meet the oral healthcare needs of older adults more effectively.
